# Developmental Social Experience Changes Behavior in a Threatening Environment in *Corydoras* Catfish

**DOI:** 10.1002/ece3.70391

**Published:** 2024-10-11

**Authors:** Munir Siddiqui, Austin Chiang, Ethan Lac, Jesse Kern, Gerald Wilkinson, Arne Jungwirth, James Allen, Riva J. Riley

**Affiliations:** ^1^ Department of Biology University of Maryland College Park Maryland USA; ^2^ Konrad Lorenz Institute of Ethology University of Veterinary Medicine Vienna Wien Austria; ^3^ Social and Public Health Sciences Unit, School of Health and Wellbeing University of Glasgow Glasgow UK

**Keywords:** behavioral ecology, behavioral evolution, *Corydoras*, developmental social experience, social behavior

## Abstract

Coordinated responses to threats are important for predator evasion in many species. This study examines the effect of developmental social experience on antipredator behavior and group cohesion in a highly gregarious catfish that communicates via tactile interaction, *Corydoras aeneus.* We reared fish either in a mixed‐age group of age‐matched peers and adult *C. aeneus* (mixed‐age condition, or MAC), or with age‐matched peers only (same‐age condition, or SAC). A startle test was conducted with small groups of subadults from either social rearing condition. Prior to any startle events, SAC subadults had increased tactile communication compared to MAC subadults, but SAC individuals were overall less active. SAC fish exhibited a stronger antipredator response to startles, and were more likely to freeze or take refuge in cover in response to a startle than MAC fish. MAC fish tended to respond to startle events by maintaining or decreasing their cohesion, whereas SAC fish tended to maintain or increase their cohesion. These behavioral differences are attributed to MAC fish developing with group protection as a result of shoaling with adults, resulting in reduced antipredator responses when reared with adults. This study underscores how social context during development can be critical in shaping how individuals perceive and respond to potential threats in their environment.

## Graphical abstract

This study examines the effect of developmental social experience on antipredator behavior and group cohesion, in a highly gregarious catfish that communicates via tactile interaction, *Corydoras aeneus*. We reared fish either in a mixed‐age group of age‐matched peers and adult *C. aeneus* (mixed‐age condition or MAC), or with age‐matched peers only (same‐age condition or SAC). SAC subadults had increased tactile communication before any startles compared to MAC subadults, were overall less active, exhibited a stronger antipredator response to startles, and were more likely to freeze or take refuge in cover in response to a startle than MAC fish.

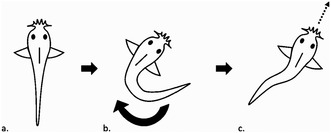

## 1 Introduction

Despite the costs of social living, such as competition for food and mates, it confers profound advantages to social animals, and in particular improved foraging and predator evasion (Ward and Webster 2016). To fully reap the benefits of social living, individuals in groups must effectively coordinate their activities with one another, as some degree of group coordination ultimately provides these benefits, and improved coordination of activities between individuals generally improves individual outcomes within a group (Sirot and Touzalin 2009). Flocks of pigeons exhibit highly coordinated responses to predators that utilize self‐organization rules to reduce the amount of attention and cognitive effort individuals must invest in predator vigilance (Papadopoulou et al. 2022). In zebra finches, synchronizing reproduction with groupmates is associated with higher reproductive success (Brandl et al. 2021). Across taxa and social contexts, modulating group coordination is essential for the evolution of social complexity (Griesemer and Shavit 2023).

Two essential aspects of effective group coordination are cohesion and communication. Cohesion is when the average distance between group members is low, and maintaining group cohesion following a threat is a powerful factor in group predator evasion in many species (Miller et al. 2013; Riley, Gillie, Savage, et al. 2019). Cohesive groups generally coordinate group movements more effectively, an effect demonstrated in multiple species including many fishes (Herbert‐Read et al. 2011). High cohesion generally provides antipredator benefits, with cohesive groups more likely to successfully evade predators (Sogard and Olla 1997; Chivers et al. 1995; Viscido and Wethey 2002). Communication between group members also facilitates group coordination and enhances group antipredator response. Some forms of group communication, such as alarm calls, involve the clear transfer of information from one individual, or a subset of a group, to the rest of the group (Schel et al. 2010), often containing specific information about the potential predator (Templeton 2005). Group communication before and during a predation threat can be influenced by a prey species' fear of predation, and in the Trinidadian guppy *Poecilia reticulata*, the perceived risk of predation caused fish to develop stronger social ties and higher group cohesion (Heathcote et al. 2017).

While communication and coordination are crucial to survival and reproduction in many species, individuals generally require developmental inputs in order to learn and express effective social coordination behaviors. In the cichlid *P. taeniatus*, group coordination is a socially developed behavior, and sub‐adults raised in isolation showed increased aggression, a delayed response to external stimuli, less shoal cohesion, and lowered growth compared to their age‐matched peers raised in groups (Hesse and Thünken 2014). In the highly social cichlid *Neolamprologus pulcher*, being raised with or without adults also affects social behavior, albeit in concert with ecological experience: fish that grew up in mixed‐age family groups in a seemingly safe environment showed the most pro‐social behavior and best integration into their social group, whereas fish that only experienced same‐age full siblings during development were less socially competent (with predator experience altering these effects; Fischer et al. 2017). The predatory mite *Phytoseiulus persimilis* exhibits a similar effect of social isolation, with mites reared in social isolation showing lower social competence and poorer reproductive outcomes (Schausberger, Gratzer, and Strodl 2017). Additionally, the absence of adult fish during development likely makes individuals worse at coordination under stress, due to fear of predation (Kelley and Magurran 2003). These studies underscore the importance of social experiences in the development of group coordination skills in individuals. Social rearing generally promotes behaviors that are beneficial for group living, such as reduced aggression, quicker responses to stimuli, and stronger group cohesion; it also suggests that isolation can lead to significant behavioral and growth deficits (Hayes and Solomon 2004; Chapman, Ward, and Krause 2008; Liedtke and Schneider 2017).

Our study species, *Corydoras aeneus, exhibits a clear developmental effect of isolation*: larvae raised in isolation exhibit lower social competence than larvae reared socially (Riley et al. 2020). In this study, we reared larvae either socially or in isolation, and then placed larvae in groups and assessed their social interactions. We found that larvae reared in isolation initiated fewer nudges with groupmates, and furthermore, were more likely to c‐start (an involuntary antipredator response) when they physically touched others. This underscores that nudging has an innate component, but nonetheless requires social experience to properly develop. This raises interesting questions about how different kinds of social experience impact social development. *Corydoras aeneus* is a promising system for investigating questions related to social development, in part because *Corydoras* catfish exhibit tactile interactions as a form of communication. These discrete tactile interactions, termed “nudges,” have been shown to facilitate coordination and cohesion in groups of fish (Riley, Gillie, Savage, et al. 2019). They are also deployed during group threat responses, in which nudges serve as a form of communication to coordinate group responses to a threat (Riley, Gillie, Savage, et al. 2019). In the wild, these fish have been observed in group sizes as small as three (Riley 2012) and live in mixed‐age, mixed‐size groups (Lambourne 1995; Riley personal observation) that form due to their extremely low levels of aggression and defensive anatomy (in contrast to many other fish systems; Hoare et al. 2000). Having a variety of sizes may make the shoal less effective in confusing predators (as confusion effects depend on group homogeneity [Krakauer 1995]), so there are likely benefits to overcome that disadvantage (Lambourne 1995; Riley 2012; Hoare et al. 2000). It is also likely that very young juveniles that hatch as a group must live in juvenile‐only shoals for some time before encountering and joining a mixed‐age shoal, since *C. aeneus* eggs are typically laid in clusters and parents do not directly provide parental care after eggs have been laid (Lambourne 1995).

This paper examines the role of social rearing condition in the environmental exploration and threat evasion effectiveness of *C. aeneus* sub‐adults, comparing those who are housed with (and thus have observed and interacted with) adult fish to those who have never had social experience with an adult and have lived entirely with age‐matched peers. The discrete, quantifiable nature of nudging behavior allows us to quantify individual and group social responses to threats, and this study will shed light on how social exposure to mature individuals impacts the development of the ecologically critical behavior of predator evasion.

We evaluated flight responses by applying a threat stimulus to groups of subadult fish and qualitatively and quantitatively evaluating their response. The first category is composed of subadult fish raised in a mixed‐age social housing condition (which we term MAC [mixed‐age condition]), and the second category is composed of subadults raised without adults and only with their peers in a same‐age social housing condition (which we term SAC [same‐age condition]). The MAC category emulates the same social environment these fish have in the wild, while the SAC category is socially deprived without the presence of adults. We define a subadult *C. aeneus* as a fish that has attained adult coloration, but is not yet sexually mature. Our previous study of *C. aeneus* larvae showed that habituation to tactile stimulus, and thus to receiving nudges, occurs starting in the larval stage, which results in a gradual decrease in flight response from tactile interactions as the fish becomes an adult (Riley et al. 2020). Accordingly, the subadult fish in this study had fully developed nudging behavior. We evaluated threat responses in subadult *C. aeneus* in two contexts: a context that is unfamiliar to the fish but is not directly threatening (termed baseline in this study), and then a second, threatening context in which fish were faced with a direct potential threat (in the form of a startle test).

We have two hypotheses about the effect of social rearing conditions on the subadult fish. The first is that the absence of adult *C. aeneus* during development makes subadult fish less socially coordinated. We would thus predict differences between the baseline behavior (i.e., behavior observed before any startle tests) in SAC and MAC groups. In particular, we expect lowered group cohesion and nudging in SAC fish, which have not been exposed to socially active adults. Second, the absence of adult *C. aeneus* during development may lead to subadult fish being unable to adequately respond to a threat. We predict that this should result in differences in group responses to threats, with SAC fish showing reduced group cohesion and increased individual‐level fear responses. Through this study, we aim to elucidate the role of mixed‐age social grouping during the development of antipredator behavior and group coordination.

## 2 Methods

### 2.1 General Husbandry

All juvenile/subadult fish in this experiment were bred from our adult stock population, housed in the Biology‐Psychology Building at the University of Maryland in College Park, Maryland, USA. Our stock population consists of wild *C. aeneus* (CW097 designation) catfish caught in the Madre de Dios region of the southern Peruvian Amazon region in October 2018. We obtained these fish in January 2021 and they have been maintained in our lab since. Our adult stock population was kept in two standard 109.78 L tanks (76.20 × 30.48 × 45.72 cm). The stocking density of these tanks was 14–15 adult fish per ~109 L aquarium. Adult fish were fed daily with Tetra brand tropical fish granules as well as twice a week with thawed frozen bloodworms (Chironomidae larvae), and water changes were performed once a week from a central RO supply remineralized with Tropic Marin Remineral Tropic to 95–100 ppm total dissolved solids (as in Riley, Gillie, Savage, et al. 2019; Riley, Gillie, Cat, et al. 2019). The stock tanks were kept in a temperature‐controlled room with a set 12:12 day/night cycle. Each tank was also equipped with a power filter (Tetra brand) and two air‐driven sponge filters, as well as sand substrate, artificial plants, and large PVC pipes for cover.

Eggs were collected from adult stock tanks in two batches, one in January 2022 and one in March 2022; both batches were collected from the same two adult stock tanks. Multiple clutches of eggs were laid by multiple sets of parents in each adult stock tank over 2 days per spawning event. Because of the unique sperm‐drinking copulation mechanism in these fish (Kohda et al. 1995), we can be confident that eggs in the same clutch (visibly distinguishable as clusters of eggs) are full siblings. On each day of the 2‐day spawning event for both batches, we put all of the eggs laid in both adult stock tanks in a separate enclosure, thereby mixing full sibling groups. This ensures that the degree of relatedness did not differ when fish were later allocated to experimental social‐rearing tanks. This also simulates natural spawning conditions, when many pairs of adults spawn around the same time and lay eggs in the same areas.

Eggs were placed in small ~22.7 L (22.23 × 50.48 × 20.32 cm) aquaria and stayed in those aquaria as larvae. Larvae were fed daily with crushed TetraColor tropical fish granules and thawed frozen baby brine shrimp, and water changes were performed every other day with the same procedure described above for the adult stock fish. After 8–9 weeks in the small aquaria (9 weeks for the first batch, 8 weeks for the second batch), larvae had developed to the juvenile stage of their life cycle and had reached a minimum standard length of 1 cm. They were then randomly placed in one of the four 56.78 L social rearing enclosures (30.23 × 46.48 × 68.58 cm), described below.

### 2.2 Experimental Husbandry

Four social rearing enclosures were used during each batch of this experiment, two SAC enclosures and two MAC enclosures, for eight total enclosures across both experimental batches. In each batch, two enclosures were randomly designated as SAC housing, and two were randomly designated as MAC housing. Juveniles were randomly allocated to enclosures to eliminate differences in the degree of relatedness between SAC and MAC enclosures. The two SAC enclosures in each batch contained 15–18 juvenile fish from the same larval‐rearing aquaria. The two MAC enclosures in each batch contained 13 juvenile fish and 5 adult *C. aeneus*. Fish were kept in the enclosures for 1 month, until they reached the subadult stage of development 90 days after hatching. The presence or absence of adult *C. aeneus* during development distinguished the two treatment categories. Fish were fed daily with TetraColor tropical fish granules and thawed frozen bloodworms twice a week. Water changes were performed twice a week from a central RO supply remineralized with Tropic Marin Remineral Tropic to 95–100 ppm total dissolved solids. The experimental enclosures were kept in a temperature‐controlled room with a set day/night cycle (the same room and parameters as the adult social‐rearing tanks described above). Each tank was also equipped with two sponge filters, and contained cover in the form of two terracotta pots to minimize stress. Additionally, a lid composed of plastic egg crate was placed on top of each aquarium to prevent fish from jumping out while allowing light in.

### 2.3 Experimental Housing

Across both batches of the experiment, we collected data from 28 groups of three juvenile fish randomly chosen from the same enclosures (so that all individuals in each group were familiar with one another) were placed in 37.85 L test tanks initially composed of half water from adult stock tanks and half remineralized RO water. Three test tanks were used simultaneously, each with the same water level and each containing one small sponge filter. The sponge filter was turned off during filming, but was left in the test tank to provide cover. Cameras were mounted above each tank at a uniform height for filming fish behavior. Each of the three tanks also had one vertically oriented lamp 8–12 in. away from the tank to ensure even lighting.

Each test tank was filmed for 2 h. Starting 30 min after filming began, a stimulus, in the form of a single tap on the glass of the test tank with one finger, was applied to each tank in 15‐min intervals. Tap tests like the one we employ in this study have been used to assess antipredator responses and responses to potential danger in other fish systems (Gotanda et al. 2012; Chanin et al. 2012). We refer to these taps as “startles” hereafter. After 2 h of filming, the fish finished the experiment. The three fish from each test tank were then placed into a social housing tank designated for fish who had completed the experiment, thus avoiding multiple testing of the same individuals. When all fish were transferred, a 50% water change in each test tank was performed using remineralized RO water. This process was then repeated for each experimental group, resulting in 28 total trials (*N*_SAC_ = 13, *N*_MAC_ = 15) that were analyzed across both batches.

We excluded groups (four groups, three SAC groups, and one MAC group) that exhibited no movement during the baseline period (described below) or experienced camera errors that prevented filming (one MAC group); so 33 groups of three fish were filmed initially.

### 2.4 Data Collection and Video Analysis

We analyzed two aspects of the videos: (1) a baseline period, defined as the 5 min before the first startle event (starting after ~25 min of filming) and (2) the behavior of the fish 3 s before and 3 s after each startle event.

For the baseline period, we scored the videos for nudges, and we collected information on the total number of nudges performed, following the protocol in Riley, Gillie, Savage, et al. (2019). This analysis includes nudges that take place off of the bottom, that is, during short periods of free swimming in the water column. The fish had 30 min to acclimate before we began the startle stimuli, and the last 5 min of this pre‐startle acclimation was scored for a baseline rate of nudges.

In addition to scoring nudges, we recorded some basic activity and sociality level information during the baseline period. We recorded the cumulative amount of time (in seconds) of all of the possible combinations of activity among the fish (no movement, one individual moving, two individuals moving, all moving), the cohesion of the fish (whether all three were apart, all three were together, or two fish were in a group separate from the third fish (Appendix 1)), and the amount of time in seconds that “wall‐surfing” behavior was observed. Cohesion was defined as being within two body lengths of another fish (Riley, Gillie, Savage, et al. 2019). Wall surfing behavior occurs when the fish move off the bottom of the tank and swim up and down the sides, and is considered a non‐standard behavior and a potential indicator of stress (Martins et al. 2012). As part of recording this information, we assumed that, as a default, all fish were actively moving, all fish were grouped together, and that wall surfing was not present. This default coordination state is their most commonly observed state in the wild (Riley, personal observation). Activity and wall surfing was measured with a 5 s threshold, where a non‐default behavior (i.e., fish were still/not together or wall surfing behavior) was only recorded if it occurred for 5 or more consecutive seconds.

To measure the cohesion of the fish, we calculated a “cohesion fish seconds” metric. To do this, we multiplied the amount of time each group spent all together (in a group size of 3) by three and the amount of time each group spent with two fish together and one apart by 2. We then added these products together for the following overall equation: (3 × all three fish together) + (2 × two fish together, one apart) = cohesion fish seconds.

To measure the activity of the fish, we calculated an “active fish seconds” metric, derived from summing the products of each possible combination times the number of fish that were moving in that combination. For example, when all three fish are moving, we multiply the amount of time they spend in that combination by 3, giving us the total number of time spent by all three fish in that configuration. We multiplied the amount of time two fish were moving by 2, and the amount of time just one fish was moving by 1. We then added these products together, for this overall equation: ([3 × all three fish moving] + [2 × two fish moving] + [one fish moving]) = active fish seconds. This takes into account the movement of every single fish, rather than just analyzing the average of each group of 3.

To measure the wall surfing, we calculated a “wall surfing fish seconds” metric in a manner similar to “active fish seconds.” We calculated this by multiplying the amount of time all three fish were wall surfing by 3, multiplying the amount of time two fish were wall surfing by 2, and multiplying the amount of time just one fish was wall surfing by 1, and then adding the products together. This takes into account the movement of every single fish to get an overall measure of wall surfing for the entire group.

**FIGURE 1:**
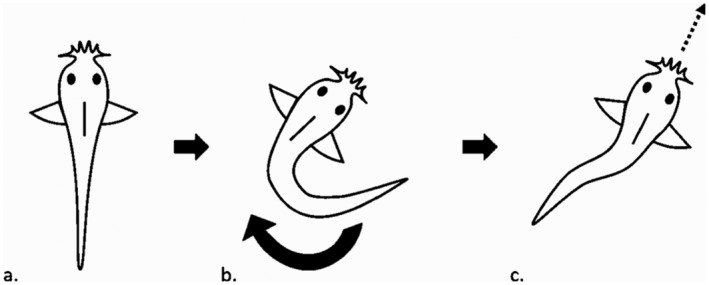
Diagram of a c‐start threat response in *Corydoras aeneus* larvae.

To score the behavioral responses to startles, we scored the same basic activity information as described above at 3 s before each startle and at 3 s after the startle. In addition, we recorded the number of active fish and the number of fish that were in cover 3 s before and after the startle, as well as how many fish moved, froze, or performed a c‐start maneuver during each startle. The c‐start is an involuntary response where the fish adopts a c shape with its body, then straightens out to travel in a direction where the fish can escape their perceived threat (Domenici and Blake 1997; see Figure 1).

### 2.5 Statistics

All data analysis was conducted in R (2022.07.2, R Core Team 2021). For continuous data, we assessed data distributions visually and used nonparametric tests when data were not normally distributed. To compare total nudges between SAC and MAC subadults, we used a Wilcoxon signed rank test. We used a two‐sample *t*‐test to compare cohesion levels between SAC and MAC fish. To compare activity levels between SAC and MAC subadults, we used a Wilcoxon signed rank test. To compare wall surfing tendencies, we also used a Wilcoxon signed rank test.

We then analyzed the group behavior associated with startle events. Because we performed four startle events, we checked for an effect of habituation on cohesion status before and after the startle, the number of c‐starts following a startle, the number of active fish before and after the startle, and the number of fish that froze in response to the startle. We used a Spearman rank correlation to assess if there was any association between each of these measures and the startle event. We performed these tests for the whole dataset (SAC and MAC fish combined), as well as for each of the two rearing conditions separately. These additional steps were included to ensure that we did not overlook potential differences between SAC and MAC fish with regard to habituation effects.

We first analyzed cohesion tendencies testing for the effects of habituation on cohesion status. We compared the number of fish in each possible cohesion status (all together, two together and one apart, all apart) three 3 s before the startle event and 3 s after the startle event in SAC and MAC fish. To assess whether MAC and SAC fish differed in their cohesion tendencies following a startle event, we used a multinomial regression using the nnet package in R; the response variables were the cohesion state after a startle event (i.e., from all fish apart, all fish together, etc.) and the explanatory variable was the cohesion state before and the social rearing condition (MAC or SAC). Startle events are expected to be experienced frequently in nature, and the time between events in our experiment is such that we expect no cumulative effect of the startle number (and found no effect of habituation). Therefore, in this analysis we treat each startle event as independent (rather than analyzing them as repeated trials). We compared models with and without the social rearing condition using the Akaike information criterion (AIC) to assess whether these transition tendencies differed between the social rearing conditions (MAC and SAC). For the full model including the social rearing condition, we found the following coefficients in a multinomial logistic regression model (where we take cohesion before = 1 as our baseline).

**Table:** 

Final cohesion	2	3
Cohesion before	0.31	9.90
House cond.	0.01	26.10
Cohesion before × House cond.	0.28	−7.98

Because we had multiple startle events for each group, we used a mixed‐effect approach to analyze the number of fish that c‐started following a scare, the number of fish that were active before and after a startle event, the number of fish in cover before and after a startle event, and the number of fish that froze before and after a startle event. Linear mixed‐effects models (LME) and generalized linear mixed‐effects models (GLMM) were fitted using the lme4 package (Bates et al. 2015). Data distributions were initially assessed visually, and model diagnostics were subsequently checked to assure appropriate fits and then verified using the DHARMa package in R (Hartig 2022).

We fitted LMEs for normally distributed data (arcsin(sqrt) transformed proportion of c‐starts in each startle event). For proportion data that could not be transformed to meet the assumptions of an LME, we fitted GLMMs with a binomial distribution. We used the optimx package (Nash and Varadhan 2011) to modify the optimizer function in binomial GLMMs when necessary to ensure model convergence.

We used a linear mixed‐effect model to compare the proportion of fish that exhibited a c‐start in each startle in MAC versus SAC fish. We used the arcsin(sqrt) transformation on the proportion of fish in a group that c‐started following a startle (number of fish that c‐started divided by 3) as the response variable. The fixed effects were the social rearing condition (MAC or SAC) and startle event (1–4). The random effect was the group ID.

We used a binomial generalized linear mixed‐effect model to compare the proportion of active fish in each group before and after a startle in MAC versus SAC fish. The proportion of fish that were active was the response variable. The fixed effects were the interaction between the social rearing condition and timepoint (before or after the startle), and startle event. The random effect was group ID. We used the optimx “L‐BFGS‐B” optimizer to ensure model convergence.

We used a binomial generalized linear mixed‐effect model to compare the proportion of fish in cover before and after a startle in MAC versus SAC fish. The proportion of fish in cover was the response variable. The fixed effects were social rearing condition, timepoint (before or after the startle), and startle event. The random effect was group ID.

We used a binomial generalized linear mixed‐effect model to compare the proportion of fish that exhibited a c‐start in each startle. We used the proportion of fish in a group that c‐started following a startle (number of fish that c‐started divided by 3) as the response variable. The fixed effects were the social rearing condition (MAC or SAC) and startle event (1–4). The random effect was the group ID.

We used the emmeans package in R (Lenth 2020) to conduct posthoc comparisons whenever an interaction between fixed effects or startle event were significant predictors in our models.

## 3 Results

### 3.1 During Baseline

#### 3.1.1 Nudges

**FIGURE 2:**
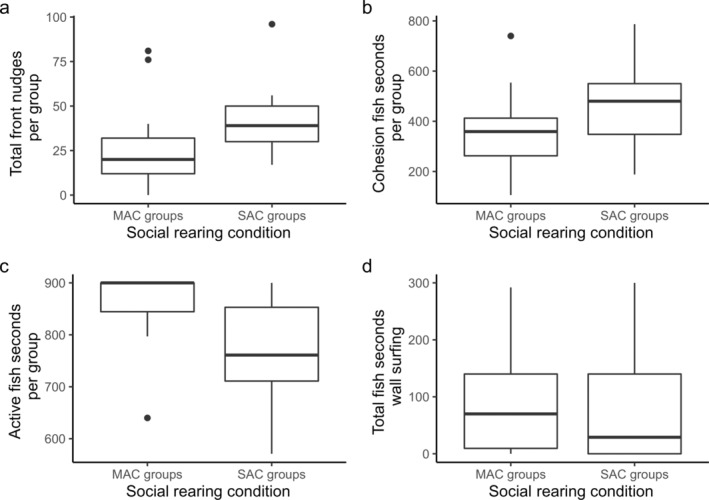
Data from the baseline period (exploration of the unfamiliar environment). (a) The number of front nudges in MAC versus SAC groups. (b) The cohesion fish seconds in MAC versus SAC groups. (c) Active fish seconds in MAC groups versus SAC groups. (d) Fish seconds wall surfing in MAC versus SAC groups. For all boxplots, the box hinges represent the interquartile range, IQR (first to third quartiles) and whiskers represent 1.5IQR.

SAC groups exhibited significantly higher rates of nudging than MAC groups (Wilcox test, *W* = 46.5, *p* = 0.020, Figure 2a).

#### 3.1.2 Coordination

##### 3.1.2.1 Cohesion

MAC fish were less cohesive than SAC fish, although this difference failed to be statistically significant (two‐sample *t*‐test, *t* = −1.9, df = 26, *p*‐value = 0.073, Figure 2b).

##### 3.1.2.2 Activity Levels

SAC groups exhibited significantly lower levels of activity than MAC groups (Wilcox test, *W* = 157, *p* = 0.005, Figure 2c).

##### 3.1.2.3 Wall Surfing

There was no difference in wall surfing tendencies based on the rearing conditions (two‐sample *t*‐test, *t* = 0.6, *p* = 0.524, Figure 2d).

### 3.2 Response to Startles

We found no association between any of the variables of interest and startle event (spearman rank correlation, all *p* > 0.20).

**FIGURE 3:**
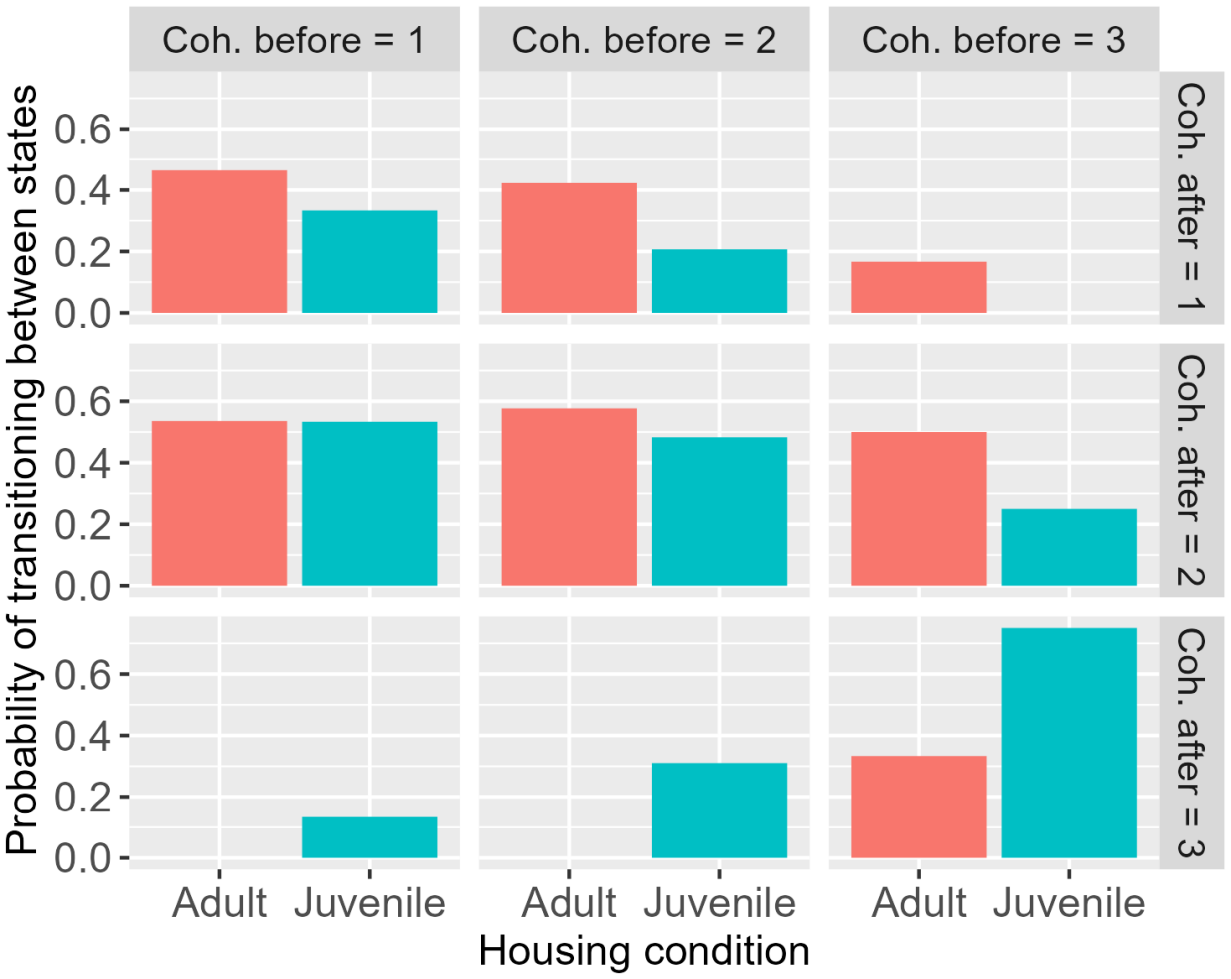
The probability of transitioning between cohesion states based on the initial cohesion state and social rearing condition.

In Figure 3, we plot the probability of transitioning between each cohesion state after a scare event. The probability is calculated as the number of those in each final cohesion state, compared to the total number that started in the cohesion state before, for example, Prob cohesion after = 2 = (Number in cohesion after = 2)/(“Number in cohesion after = 1” + “Number in cohesion after = 2” + “Number in cohesion after = 3”). These are compared for each social rearing condition.

Including social rearing condition (2 level factor: SAC or MAC) in the model of changes to group cohesion in response to startles improved model fit (AIC = 202 vs. AIC = 215). This difference in AIC of 13 provides evidence that a model without housing condition has essentially no support (Burnham and Anderson 2004). SAC fish generally tended to improve cohesion in response to a startle (Table 1; 19/52 instances of increasing cohesion), whereas MAC fish tended to reduce it (Table 2; 15/60 instances of reducing cohesion). The same number of MAC groups increased cohesion as decreased (15 and 15), but more SAC groups increased cohesion than decreased (19 vs. 8). Table entries labeled colored blue represent groups that exhibited greater cohesion after the startle than before, while entries colored red represent groups that exhibited lesser cohesion after the startle than before.

**TABLE 1:** Cohesion transitions of groups in SAC fish 3 s before and after startle events.

	After the Startle		
	All apart	Two together, one apart	All together
**Before the Startle**			
All apart	5	8	2
Two together, one apart	6	14	9
All together	0	2	6

*Note:* Purple cells indicate the number of groups that maintained a certain level of cohesion before/after startles, red cells indicate groups that decreased cohesion levels, and blue cells represent groups that increased cohesion levels.

**TABLE 2:** Cohesion transitions of groups in MAC fish 3 s before and after startle events.

	After the Startle		
	All apart	Two together, one apart	All together
**Before the Startle**			
All apart	13	15	0
Two together, one apart	11	15	0
All together	1	3	2

*Note:* Purple cells indicate the number of groups that maintained a certain level of cohesion before/after startles, red cells indicate groups that decreased cohesion levels, and blue cells represent groups that increased cohesion levels.

**FIGURE 4:**
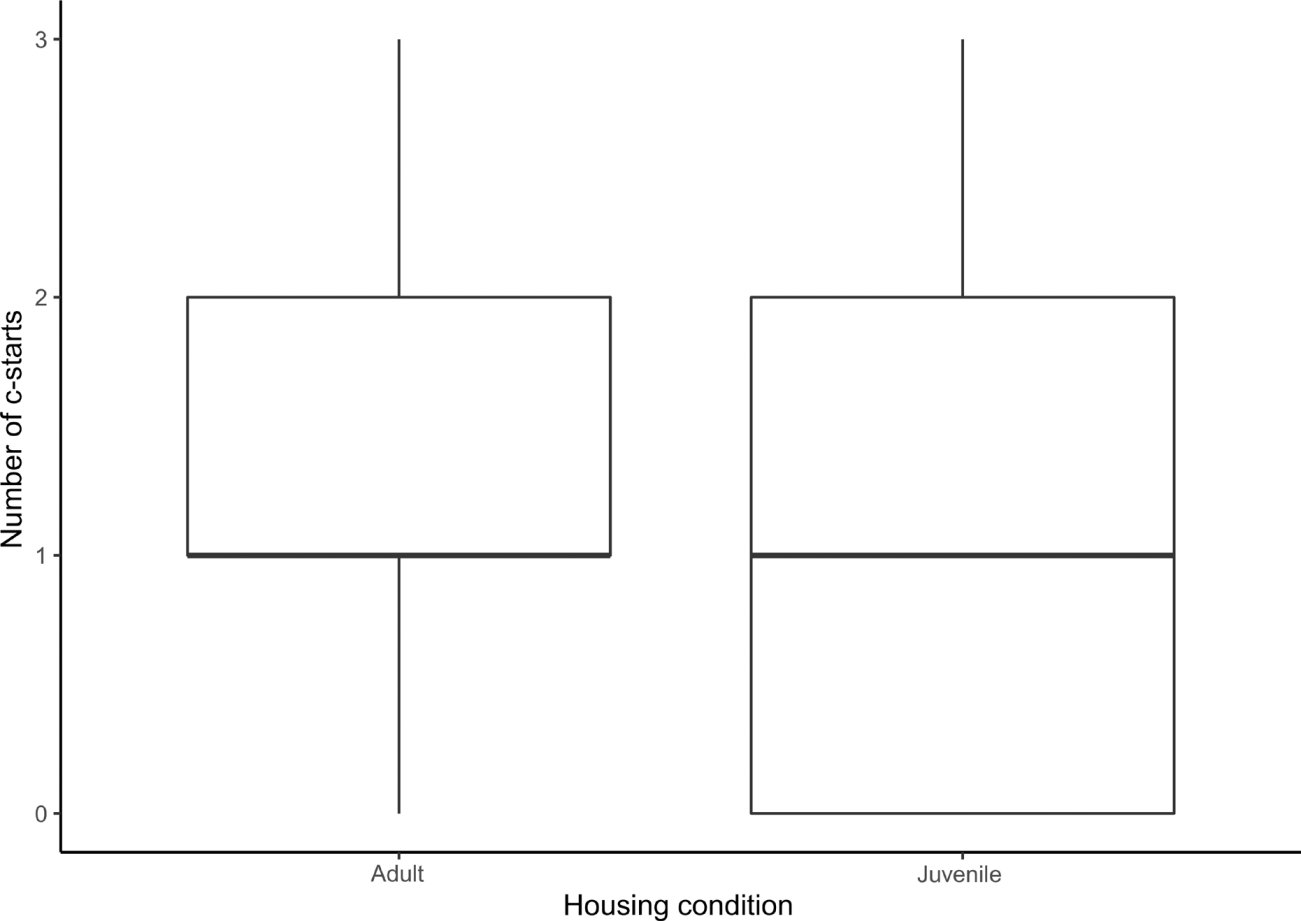
The number of c‐start responses at each startle for MAC and SAC groups.

Neither social rearing condition (*F* = 1.2, *p* = 0.278) nor startle event (*F* = 0.97, *p* = 0.327) were significant predictors of the proportion of fish in a group that exhibited a c‐start response to a threat event (see Figure 4).

**FIGURE 5:**
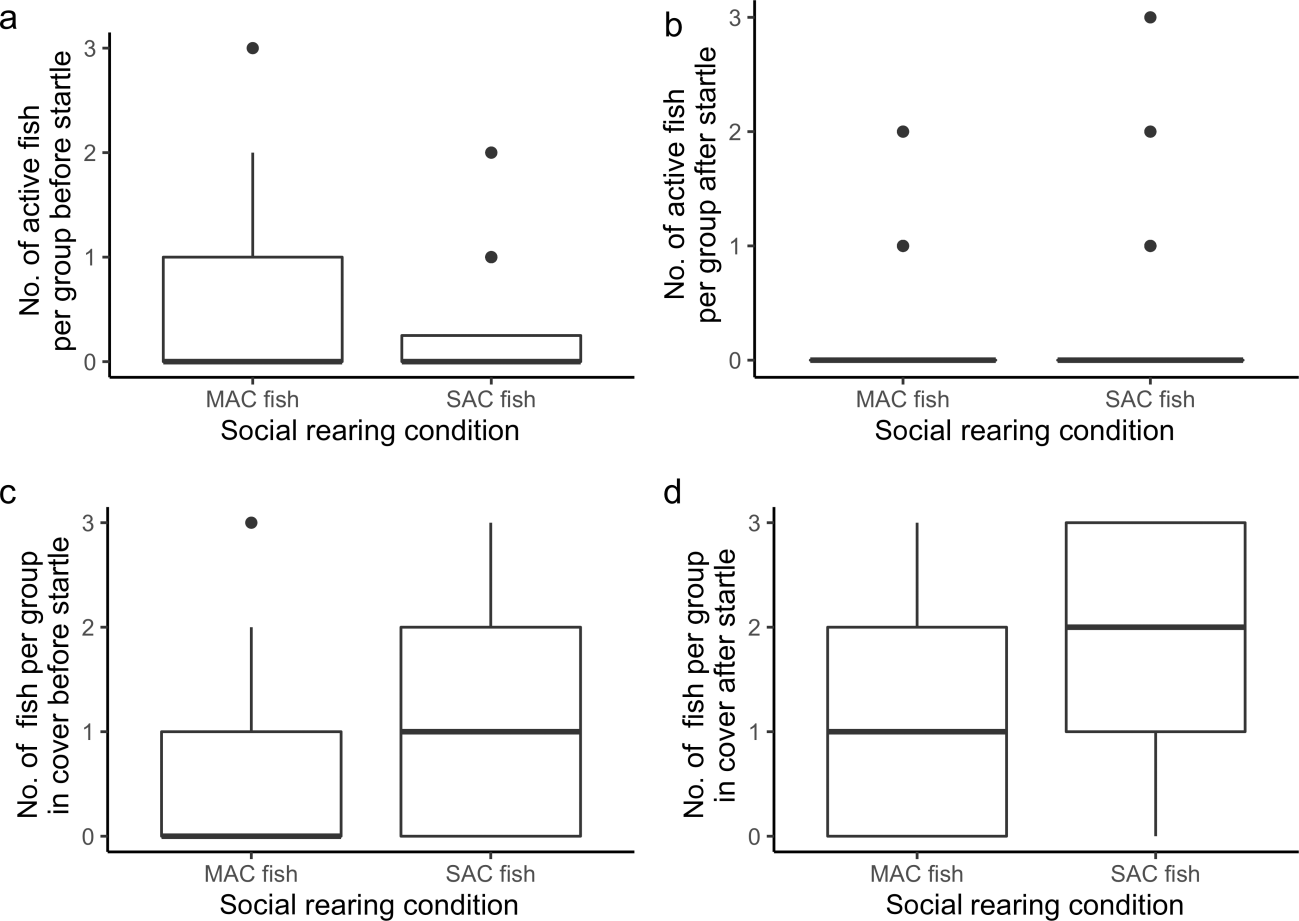
(a, b) Number of fish per group, either SAC or MAC condition, that were active 3 s before (a) and after (b) a startle. (c, d) The number of fish per group, either SAC or MAC condition, that were in cover both before (c) and after (d) a startle event.

Both startle event (LRT = 8.4, *p* = 0.038) and the interaction between the social rearing condition and timepoint (LRT = 5.2, *p* = 0.022) were significant predictors of the number of active fish per group. Posthoc analysis shows that MAC fish exhibited a low number of fish that were active both before and after a scare, whereas SAC fish were significantly more active before a startle than after a startle (see Figure 5a,b). The number of active fish per group per startle event significantly differed between startles 2 and 3, but not any other combination. Social rearing (LRT = 10, *p* = 0.002), timepoint (LRT = 31.8, *p* < 0.0001), and startle event (LRT = 5.4, *p* = 0.021) were all significant predictors of the number of fish in cover (see Figure 5c,d). The number of fish in cover per startle event significantly differed between startles 1 and 2 and 1 and 3, but not any other combination.

**FIGURE 6:**
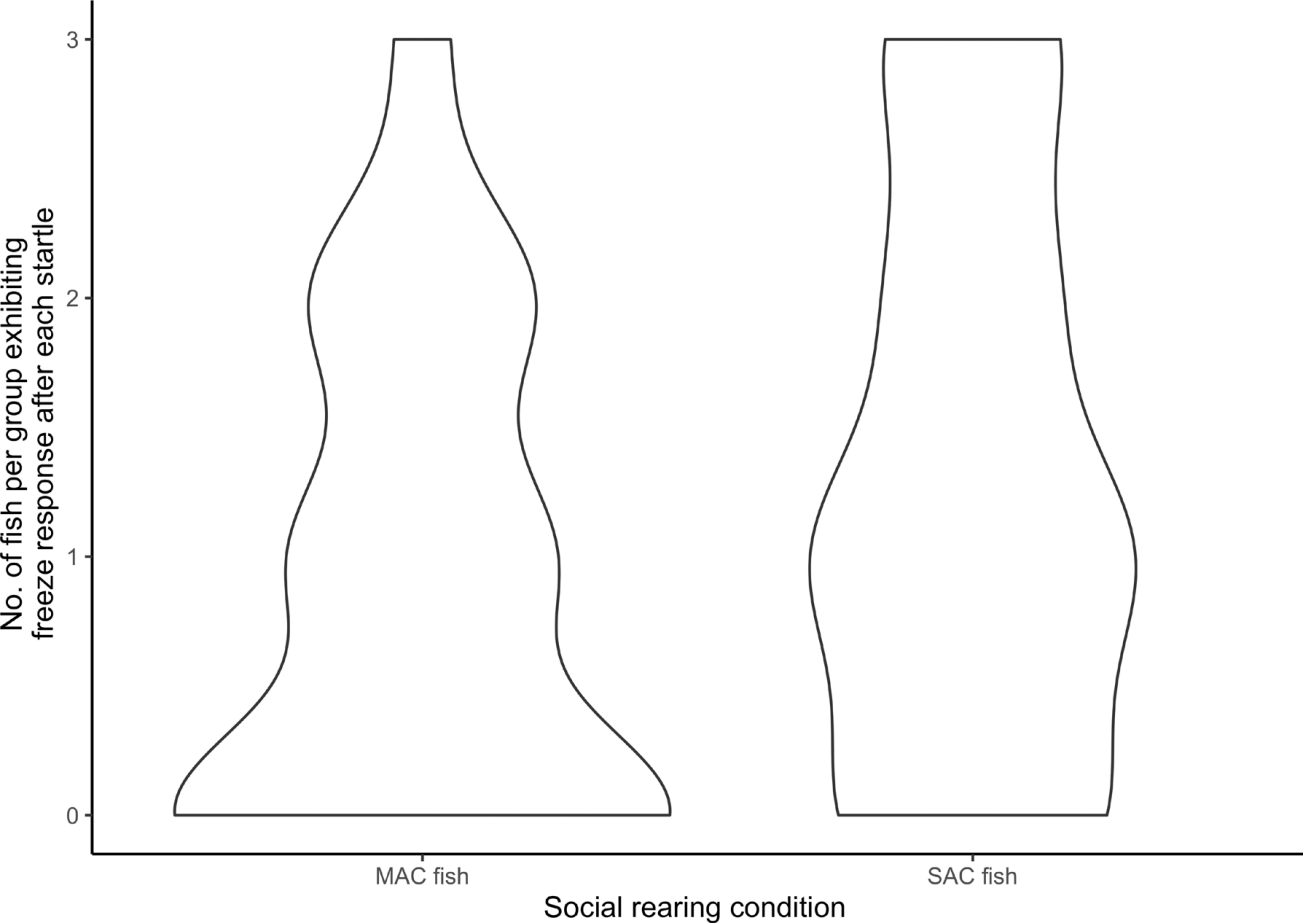
The number of fish per group, either SAC or MAC condition, that exhibited a freeze response after a startle event.

Startle event was not a significant predictor of the proportion of fish that exhibited a freeze response (LRT = 2.3, *p* = 0.130). Social rearing condition, however, was a significant predictor (LRT = 4.2, *p* = 0.041, Figure 6).

## 4 Discussion

Our results show that at a baseline (i.e., when fish were exploring an unfamiliar environment prior to any startle events), SAC subadults have increased nudging behavior compared to their MAC peers, but are less active. In terms of startle events, we saw no effect of habituation across the four startle events in this experiment: all correlations between our response variables and startle event were not significant, and in models where startle event was a significant predictor of the response variable, there was no consistent trend that indicates habituation (i.e., the differences between startle events seemed to be random, and not a systematic change as startle events progressed) and SAC fish were more likely to freeze after a startle than their MAC peers. Additionally, group cohesion before and after four startle events was significantly different, with SAC fish increasing cohesion more than MAC fish when being startled. SAC fish also exhibited a higher degree of antipredator behavior in response to startle events, were more likely to be in cover both before and after a startle, as well as to freeze in response to startles, which is a common antipredator behavior in these fish (Riley, Gillie, Savage, et al. 2019). These results ran counter to our original hypotheses, with the SAC individuals being more interactive and cautious than MAC individuals, despite being socially deprived of interactions with adult fish. These unexpected findings highlight the diverse ways developmental experience modifies behavior, with important ecological consequences.

Our hypothesis that MAC fish would exhibit heightened antipredator responses stems from the fact that young animals living in mixed‐age groups often acquire critical life skills from observing adults (van Schaik 2010). The locality that our study population originates does contain several predator species (Riley, 2012), and this population of fish has a variety of behavioral responses to predation pressure. Accordingly, we hypothesized that juvenile *C. aeneus* would learn antipredator behaviors (including surveillance) from observing adult groupmates. Our results that MAC fish exhibited a lower degree of caution and responses to startle seem to run counter to this, but nonetheless, both phases of our study indicate that the behavioral development of young fish is influenced by the presence of adult conspecifics. The effect we observe is that, instead of acquiring enhanced antipredator skills, adult groupmates modify the behavior of young fish by seemingly relaxing their antipredator responses.

The behavior of fish during baseline indicates that SAC fish are more cautious than their MAC peers. SAC individuals exhibited heightened nudging behavior compared to their MAC peers, despite markedly lower activity levels: this implies that SAC individuals devote much more of their active time to nudging. As nudging facilitates social coordination in these fish (Riley, Gillie, Cat, et al. 2019), our results support the idea that SAC fish devote more energy to social coordination and less to exploration. In addition, the lowered activity of SAC fish suggests heightened attention to minimizing conspicuousness: *C. aeneus* often deploys a freeze response to take advantage of their cryptic coloration to escape detection (Riley, Gillie, Savage, et al. 2019), and freezing and lowered activity as an antipredator response strategy has been shown to be present in other systems such as black carp *Mylopharyngodon piceus* (Tang et al. 2017). An additional reason for the decreased activity among SAC fish may be attributed to predation pressure leading prey species to conserve energy for predator detection, an effect seen in other species, including Brown Trout (Bachman 1984). MAC fish may not exhibit this energy‐conserving pattern of behavior due to the group security that they experienced during development: adults maintaining vigilance within a shoal setting may allow young fish in that shoal to forage without the need to reduce activity to devote energy to antipredator vigilance, a phenomenon that has been observed in meerkats (Santema and Clutton‐Brock 2013). This is consistent with previous studies that show that small individuals benefit from shoaling with larger conspecifics in many species, including zebrafish (Aslanzadeh et al. 2019) and prairie voles (Solomon 1993). In addition to their larger size, adult *C. aeneus* have fully developed armor and locking, venomous spines, which may further deter predators and offer protection to more vulnerable juveniles (Riley, 2012). The *Corydoras* genus also exhibits Mullerian co‐mimicry between related species, which suggests that predators may learn to recognize that *Corydoras* catfish are generally poor prey items, and juveniles benefit from associating with larger *Corydoras*, which are more easily perceived and avoided (Alexandrou et al. 2011). Given that *Corydoras* species do not exhibit aggressive behavior (Riley, 2012), the costs to juveniles of associating with older, larger fish are limited, further emphasizing the benefits to young fish of shoaling with more visible adults, whose appearance may deter predators and provide protection to juveniles and subadults that can then forage more freely.

These benefits also shed light on the higher risk‐taking of MAC fish and higher caution of SAC fish during the startle events. MAC fish were less likely than SAC fish to be in cover both before and after startle events, and SAC fish were similarly less likely to be active before the startle and more likely to freeze in response to the startle. Given that freezing is a common antipredator response in these fish (Riley, Gillie, Savage, et al. 2019), we conclude that the SAC fish are both more cautious during exploration and more likely to adopt behaviors to be less conspicuous to predators in response to a startle. This is also supported by the result that SAC fish tended to remain in cover before a startle. Without the direct perception of a threat, the tendency of SAC fish to remain in the cover is suggestive of a higher degree of cautious behavior, as is their tendency to increase their cohesion, given that increasing cohesion is a common response to potential threats across taxa (Brown et al. 2001; Viscido and Wethey 2002). For SAC fish, upregulating nudging and downregulating activity may be a more advantageous way of balancing the risks of predation versus the benefits of active social coordination. For MAC subadults, the benefits of larger, more experienced conspecifics may lead to the upregulating of exploration and foraging behaviors, instead of increased social transmission of antipredator behaviors. Our previous work showed that nudging is an innate behavior in these fish, but is strongly influenced by social conditions during development (Riley et al. 2020); accordingly, the SAC fish's increased nudging during baseline and cohesion during startle events suggests that antipredator behaviors have a substantial innate component, but individual behavioral patterns are influenced by social development.

While the effect of the social rearing condition is apparent in both the baseline and startle events, our results demonstrate that the social rearing condition did not cause pathological levels of stress or aberrant behaviors in either condition during either phase of our study. The lack of a significant difference in wall‐surfing behavior during baseline between rearing conditions supports that neither MAC nor SAC fish exhibited higher levels of stress in response to a novel environment, but rather that SAC fish were responding with increased caution. Furthermore, the lack of difference in c‐start frequency during startle events between MAC and SAC fish implies that SAC fish are not inherently more reactive to potential threats. These commonalities in wall‐surfing and c‐start frequency show that the observed differences between SAC and MAC fish reflect a difference in caution and antipredator responses, and not that SAC fish were adversely affected by the experimental design.

The results discussed above shed light on how a species' social behavior can profoundly affect its ecology. For *Corydoras* catfish, the combination of extremely low levels of aggression and defensive anatomy in adults apparently leads to strong benefits from mixed‐age, mixed‐size shoals (in contrast to many other fish systems; Hoare et al. 2000), which likely accrue in direct (to juveniles, who receive protection and improved foraging) and indirect (an adult's offspring benefit from the presence of adults) ways. By understanding how these fish adjust their behavior based on the relative vulnerability of their shoals, we can gain insights into their survival strategies and their ability to adapt to changing environmental conditions. While fish live in mixed‐age shoals in the wild, it is very much in the realm of possibility that very young juveniles hatch as a group and live in a juvenile‐only shoal for some time before encountering and joining a mixed‐age shoal, since *C. aeneus* eggs are typically laid in clusters and parents do not directly provide parental care (Lambourne 1995). Consequently, adjusting behavior based on the relative vulnerability of juvenile‐only or subadult‐only groups compared to mixed‐age groups, where the experience and physical presence of larger adults confers protection to younger fish, is certainly adaptive. Continued assessment of the role of social learning on the development of nudge coordination and group shoaling behavior in *C. aeneus* would add insight into the ecodevelopmental effects of social behavior. Further work focusing on the behavior of socially deprived individuals (i.e., individuals who have had highly constrained social experience) can help us understand the effects of social deprivation on behavior and survival. This is particularly relevant in the context of habitat fragmentation or population declines, where individuals may be more likely to experience social isolation.

Further work on how adults and juveniles interact would shed light on the behavioral mechanisms that drive the increased exploration and lower response to potential threats in MAC fish. *Corydoras* is an extremely promising system for investigating the developmental effects of the social rearing environment, and furthermore, how social ecology can drive behavioral evolution.

## Author Contributions

**Munir Siddiqui:** conceptualization (lead), data curation (equal), formal analysis (supporting), investigation (lead), methodology (lead), supervision (supporting), visualization (supporting), writing – original draft (equal), writing – review and editing (equal). **Austin Chiang:** conceptualization (supporting), investigation (equal), methodology (equal). **Ethan Lac:** data curation (equal), methodology (supporting), writing – original draft (supporting), writing – review and editing (supporting). **Jesse Kern:** conceptualization (supporting), investigation (equal), methodology (supporting). **Gerald Wilkinson:** funding acquisition (supporting), supervision (supporting), writing – original draft (supporting), writing – review and editing (supporting). **Arne Jungwirth:** conceptualization (supporting), data curation (supporting), formal analysis (supporting), methodology (supporting), writing – original draft (equal), writing – review and editing (supporting). **James Allen:** data curation (supporting), formal analysis (lead), writing – original draft (supporting), writing – review and editing (supporting). **Riva J. Riley:** conceptualization (equal), data curation (lead), formal analysis (lead), funding acquisition (lead), investigation (equal), methodology (equal), project administration (lead), supervision (lead), visualization (lead), writing – original draft (equal), writing – review and editing (equal).

## Conflicts of Interest

The authors declare no conflicts of interest.

## Figures and Tables

**FIGURE A1:**
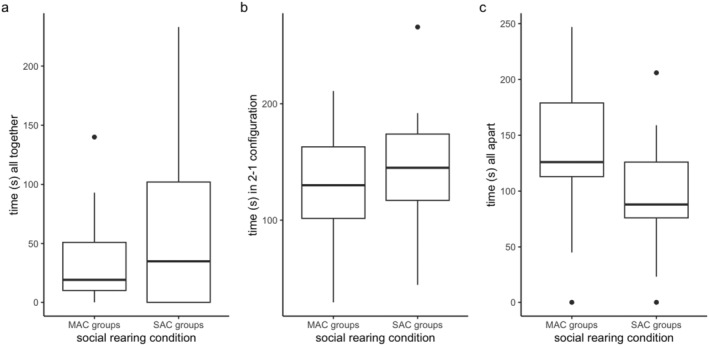
Time in all possible cohesion configurations in MAC versus SAC fish.

## Data Availability

All data, as well as the R code we used in our multinomial model, are stored at Dryad and can be found here: https://datadryad.org/stash/share/gKyMxMASkICCiiqqcwYvtitCKnja‐QPulNmUR8yECP0.

## Appendix 1

### 1.1

Our video scoring of cohesion involves three different cohesion configurations possible for a group of three individuals. Namely, the group can have all individuals together (all together), have two individuals together and one individual apart (2–1 configuration), or all individuals apart (all apart). These configurations are mutually exclusive. When analyzing all three configurations separately, we see no significant differences in the amount of time spent in each configuration (two‐sample *t*‐tests, all *p* > 0.05, Figure A1).
