# Correction to Developmental Social Experience Changes Behavior in a Threatening Environment in Corydoras Catfish

**DOI:** 10.1002/ece3.70617

**Published:** 2024-12-10

Siddiqui, M., A. Chiang, E. Lac, J. Kern, G. Wilkinson, A. Jungwirth, J. Allen, and R. Riley. 2024. “Developmental Social Experience Changes Behavior in a Threatening Environment in Corydoras Catfish.” *Ecology and Evolution* 14: e70391. https://doi.org/10.1002/ece3.70391.

In the above paper, the funding information for J. Allen was missed. We therefore include the phrase “JA was funded for part of this work by the Medical Research Council (MC_UU_00022/3)” in both the Funding and Acknowledgments sections.

We apologize for this error.

